# Wet-Spun Graphene-Enhanced PVDF Fibers for Flexible Nanocomposites

**DOI:** 10.3390/ma19071376

**Published:** 2026-03-30

**Authors:** Susanna Vu, Kablan Ebah, Fatma Zaibi, Abouelkacem Qaiss, Mohamed Siaj, Ricardo Izquierdo

**Affiliations:** 1 Department of Electrical Engineering, École de Technologie Supérieure, 1100 Rue Notre-Dame Ouest, Montréal, QC H3C 1K3, Canada; 2 Department of Chemical Engineering and Biotechnological Engineering, Université de Sherbrooke, 2500 Boulevard de l’Université, Sherbrooke, QC J1K 2R1, Canada; mohamed.siaj@usherbrooke.ca; 3 Materials Science, Energy and Nanoengineering Department (MSN), Mohammed VI Polytechnic University (UM6P), Lot 660, Hay Moulay Rachid, Ben Guerir 43150, Moroccoabouelkacem.qaiss@um6p.ma (A.Q.)

**Keywords:** graphene, fiber, wet-spinning, nanocomposite

## Abstract

Graphene incorporation into polymer fibers offers a strategy to tune nanoscale morphology while preserving mechanical conformity for flexible composite applications. Graphene-based dopants can enable modulation of polymer fiber structure; however, the relationship between graphene incorporation, fiber morphology, and mechanical flexibility must be evaluated. This study investigates the integration of graphene oxide (GO) and reduced graphene oxide (RGO) into fibrous materials to tailor the structural and surface characteristics by fabricating GO- and RGO-enhanced poly(vinylidene fluoride) (PVDF) fibers via a wet-spinning process and examining the tunability of their morphology and its influence on mechanical properties. The effect of graphene doping and reduction state on fiber architecture is explored using scanning electron microscopy (SEM), atomic force microscopy (AFM), and Brunauer–Emmett–Teller (BET) surface area analysis. Fourier transform infrared (FTIR) and Raman spectroscopy analyses confirmed the incorporation and reduction of graphene derivatives within the PVDF matrix while revealing corresponding changes in chemical functionality and the piezoelectric phase of PVDF. Mechanical flexibility is assessed through tensile testing, revealing increased stiffness with graphene addition, although maintaining sufficient structural integrity for wearable applications. These results collectively demonstrate that graphene doping provides a facile route to engineer composite fibers, enabling a balance between morphological complexity and mechanical compliancy, while establishing graphene-enhanced fibers as promising materials for flexible sensing systems and wearable smart textiles.

## 1. Introduction

Functional fibers have become sought-after materials due to interest in wearable technologies, which demand materials that are structurally complex while exhibiting mechanical integrity [1,2]. Fibrous architectures are particularly well-suited for electronic textiles and wearable devices, as they offer inherent flexibility, conformability, and compactability, allowing convenient integration into clothing and contact with skin [3,4,5]. The rising relevance of wearable technology and smart clothing in healthcare, sports, and defense industries is reflected in the global e-textile market, accounting for USD 4.3 billion in 2026 and projected to increase to USD 7.8 billion by 2034 [6,7]. Advancement in this growing field is achievable through the design of innovative flexible materials for wearable electronics [8,9].

Precise control of fiber morphology at the micro- and nanoscale is crucial for designing high-performance fibrous materials for wearable electronics [10,11]. Structural features, including shape, size, and domain distribution, can govern the performance of nanocomposites by affecting the nanomechanical behavior, such as stiffness, strength, modulus, and elasticity [12,13,14,15]. Consequently, morphology manipulation of fibers can lead to optimization of the structure–property relationship, allowing for tunability of desirable effects, namely, robustness or piezoelectricity, which are valuable for industrial applications [16].

Graphene-based nanomaterials have emerged as promising dopants for polymer fibers owing to their exceptional mechanical properties, large specific surface area, and chemical stability [17,18,19]. Among these materials, graphene oxide (GO) and reduced graphene oxide (RGO) are especially attractive due to their tunable surface chemistry and good compatibility with a wide range of polymer matrices [2,18]. Poly(vinylidene fluoride) (PVDF) is a versatile polymer with piezoelectric properties, widely exploited for its flexibility and chemical inertness, along with its compatibility with scalable fiber fabrication techniques such as wet-spinning [20]. While previous studies on graphene–PVDF composites have predominantly focused on electrospun systems and emphasized electrical [21,22] or thermal properties [23,24], comparatively limited attention has been devoted to the investigation of surface morphology of wet-spun fibers and its influence on mechanical performance in composite fiber systems. Further study of this important relationship is warranted to enable the rational design of graphene-enhanced fibers with highly tunable structure and properties.

In this study, the effects of GO and RGO content on the morphological and mechanical properties of fibrous composites were investigated. The fabrication of GO- and RGO-loaded PVDF-based continuous fibers was achieved through a simple and scalable wet-spinning process. Optical microscopy (OM) and scanning electron microscopy (SEM) were used to explore the morphology evolution upon the introduction of GO and RGO, examining the fiber microstructures and surface defects. Atomic force microscopy (AFM) expanded on fiber surface morphology by revealing the surface topology and roughness, while Brunauer–Emmett–Teller (BET) analysis quantified the surface area of the fibers. Fourier transform infrared (FTIR) spectroscopy was employed to assess the chemical composition, as well as provide insight into the potential piezoelectric characteristics of the fibrous material. The mechanical properties were characterized through tensile testing to evaluate tensile strength, Young’s modulus, and strain at yield. These results demonstrate that graphene loading and reduction provide a strategic platform for tuning the morphology and mechanical properties of fibers while preserving their functionality and flexibility. These findings allow for the design of graphene-enhanced fiber materials with potential applications for advanced wearable electronics.

## 2. Materials and Methods

### 2.1. Materials

Graphite flakes and polyvinylidene difluoride were obtained from Sigma Aldrich (Oakville, ON, Canada). Potassium permanganate was acquired from Anachemia (Lachine, QC, Canada), while sulfuric acid and phosphoric acid were received from Caledon Laboratory Chemicals (Georgetown, ON, Canada). Reagents and solvents, including hydrogen peroxide, hydrochloric acid, ethanol, and dimethylformamide, were obtained from Fisher Scientific (St-Laurent, QC, Canada).

### 2.2. Preparation of GO

Graphene oxide was prepared using a modified Hummers’ method [25]. Graphite flakes (3.0 g, 0.25 mol, 1 wt.% equivalent) were combined with H_2_SO_4_ (360 mL, 6.7 mol). To the mixture, H_3_PO_4_ (40 mL, 0.77 mol) was added slowly, and then stirred at 55 °C for 1 h. The mixture was removed from the heat and placed in an ice bath while stirring. While maintaining the temperature below 40 °C, KMnO_4_ (18 g, 0.11 mol, 6 wt.% equivalent) was added slowly in six equal portions. The mixture underwent two repetitions of ultrasonication for 30 min and stirring at 55 °C for 2 h. The reaction was subsequently ultrasonicated for 30 min and stirred at 60 °C for 48 h, with additional ultrasonication at the 24-h timepoint. The reaction was allowed to cool to room temperature, then neutralized with a mixture of ice water (600 mL) and H_2_O_2_ (10 mL, 0.43 mol). The mixture was stirred at room temperature for 1 h. The reaction workup involved sifting the mixture through a metal sieve and filtering using a glass-fritted funnel. The filtrate was centrifuged at 10,000 rpm in 16 °C for 2 h, and the supernatant was decanted and discarded. The resulting solid pellet was diluted with deionized water (800 mL), then probe-sonicated in 15-min intervals until homogeneity was achieved. Following additional centrifugation, the pellet was recovered, and the supernatant was disposed of. The solid was put through a washing process where centrifuging once with HCl (30%, 200 mL) and twice with EtOH (100%, 400 mL) were carried out. For each wash, the mixture was sonicated and filtered; then the solid was recovered from centrifugation set to 10,000 rpm at 16 °C for 2 h. The remaining solid was coagulated with Et_2_O (500 mL), and the resulting suspension was filtered. The material was finally dried through lyophilization to obtain GO flakes (yield: 4.8 g).

### 2.3. Wet-Spinning of GO-PVDF Composite Fibers

In preparing solutions for wet-spinning, the PVDF concentration is maintained at 30 wt.%, while the GO concentration is adjusted to 1 wt.%, 2 wt.%, and 3 wt.% to provide varied GO loading. To prepare the control solution, PVDF (1.5 g) was dissolved in DMF (3.50 g, 3.70 mL) by stirring at 50 °C for 5 h. To prepare a 1 wt.% GO-PVDF solution, the following procedure was used: (1) PVDF (1.5 g) was dissolved in DMF (2.45 g, 2.60 mL) by stirring at 50 °C for 5 h; (2) GO (50 mg) was dispersed in DMF (1 g, 1.06 mL) and probe-ultrasonicated for 1 h in 15-min intervals at 70% amplitude; (3) the GO solution was combined with the PVDF solution, followed by stirring at room temperature for 1 h and bath-sonicating for an additional 1 h to ensure homogenous mixing. To prepare 2 wt.% and 3 wt.% GO-PVDF solutions, steps (1) and (2) were repeated using the corresponding PVDF and GO masses as specified in Table 1. Step (3) was carried out for all solutions.

The wet-spinning set-up was specifically designed for these experiments and included a syringe pump, coagulation bath, and fiber collector, and was equipped with a Keithley 2420 Source Meter Unit (Keithley Instruments, Cleveland, OH, USA). The wet-spinning process involved loading the prepared GO-PVDF solution into a syringe connected to the syringe pump set to dispense at a controlled rate of 1 mL/min. The solution was extruded through a 22-gauge needle spinneret and injected into a coagulation bath containing 1 M NaOH at room temperature. The resulting fibers were continuously collected on a rotating cylindrical drum with an 8 cm diameter at the rate of 30 rpm (approx. 7.5 m/min). The wet-spun GO-PVDF fibers were dried at 80 °C for 12 h to remove residual solvent prior to characterization or stored in deionized water in preparation for subsequent reduction.

### 2.4. Fabrication of RGO-PVDF Fibers

To obtain RGO, the GO was chemically reduced with hydrazine (35 wt.% in H_2_O) [26]. Reduction of wet-spun fibers was carried out by cutting the thread-like GO-PVDF fiber to 300 mg, followed by submerging the fiber material into 10 mL of a mixture containing 9 μL/mL hydrazine in deionized water. The fibers were stirred in the solution and heated at 80 °C for 12 h. The fibers were subsequently removed from the solution and subjected to a thermal treatment at 80 °C under vacuum for an additional 12 h.

### 2.5. Characterization

#### 2.5.1. Structural Properties

The microstructure and morphology of the graphene-enhanced fibers were investigated using optical microscopy (OM, Keyence VHX, Mississauga, ON, Canada) and scanning electron microscopy (SEM, Hitachi TM, Tokyo, Japan), employing a 15 kV accelerating voltage.

Atomic force microscopy (AFM, Bruker MultiMode 8 with NanoScope V, Billerica, MA, USA) was used to characterize the nanoscale surface topography and quantify the surface roughness of the fibers. Contact mode was operated with a silicon tip on a nitride lever probe. AFM measurements were performed with a scan size of 10 μm × 10 μm and a scan rate of 0.98 Hz. At least three scans were measured for each formulation.

A Brunauer–Emmett–Teller (BET, TMAXCN, Xiamen, China) system was used to analyze the specific surface area of the fibers and assess the influence of graphene incorporation and reduction on surface accessibility. For each BET measurement, 500 mg of fibers were used for each formulation to determine the specific surface area.

Fourier transform infrared spectroscopy (FTIR, PerkinElmer, Waltham, MA, USA) was used in attenuated total reflection (ATR) mode, in the spectral range 4000–400 cm^−1^, to identify the chemical compositions of the samples prepared and the various interactions present.

In order to analyze quantitatively the relative β-phase content of PVDF, the characteristic FTIR absorption spectra at 765 cm^−1^ and 840 cm^−1^ were chosen to assess the respective α- and β-phase contents in each sample. The β-phase content was determined assuming that infrared absorbance obeys Lambert–Beer’s law, as expressed by the following formula:

(1) $$F(\beta )=\frac{A_{\beta }}{(K_{\beta }/K_{\alpha }{)A}_{\alpha }+A_{\beta }}\times 100\%$$

where $A_{\alpha }$ and $A_{\beta }$ correspond to the absorbance of the *α* (765 cm^−1^) and *β* phase (840 cm^−1^) in PVDF, respectively. $K_{\alpha }$ and $K_{\beta }$ are the absorption coefficients for their respective wavenumbers ($K_{\alpha }$ = 6.1 × 10^4^, $K_{\beta }$ = 7.7 × 10^4^ cm^2^ mol^−1^).

Raman spectroscopy (Renishaw inVia, Wotton-under-Edge, UK) was used to evaluate the structural characteristics and evolution of defects, accompanied with a near-infrared diode 785-nm excitation wavelength.

#### 2.5.2. Mechanical Testing

Tensile tests were performed at room temperature on a testing machine (Tinius Olsen, Horsham, PA, USA), model H10KT, according to ASTM D638 [27], at a displacement speed of 10 mm/min, employing a 5 kN load cell. At least three produced samples were tested for each formulation. The average values of parameters such as Young’s modulus (*E*), tensile strength ($\sigma_{max}$), and strain at yield ($\varepsilon_{y}$) are presented along with their standard deviations. The mechanical parameters were calculated using the equations below:

Tensile strength ($\sigma_{max}$):

$$\sigma_{max}=\frac{F_{max}}{A_{0}}$$

where $F_{max}(N)$ is the maximum force before failure and $A_{0}({mm}^{2})$ is the initial cross-sectional area.

Young’s modulus (E):

$$E=\frac{d\sigma }{d\varepsilon }$$

determined from the slope of the initial linear elastic region of the stress–strain curve.

Strain at yield ($\varepsilon_{y}$):

$$\varepsilon_{y}=\frac{{∆L}_{y}}{L_{0}}$$

where $L_{y}$ and $L_{0}$ represent the length at the elastic limit and the gauge length.

#### 2.5.3. Statistical Analysis

Statistical analysis was performed using OriginPro software (version 2017, OriginLab Corporation, Northampton, MA, USA). A two-way analysis of variance (ANOVA) was carried out with a 95% confidence level to evaluate the influence of load type (GO and RGO) and load content (1–3 wt.%) on tensile parameters. The F-statistics were determined as the ratio of the mean square between groups to the mean square within groups, and a *p* < 0.05 was deemed statistically significant.

## 3. Results and Discussion

### 3.1. Fiber Fabrication

**Figure 1:**
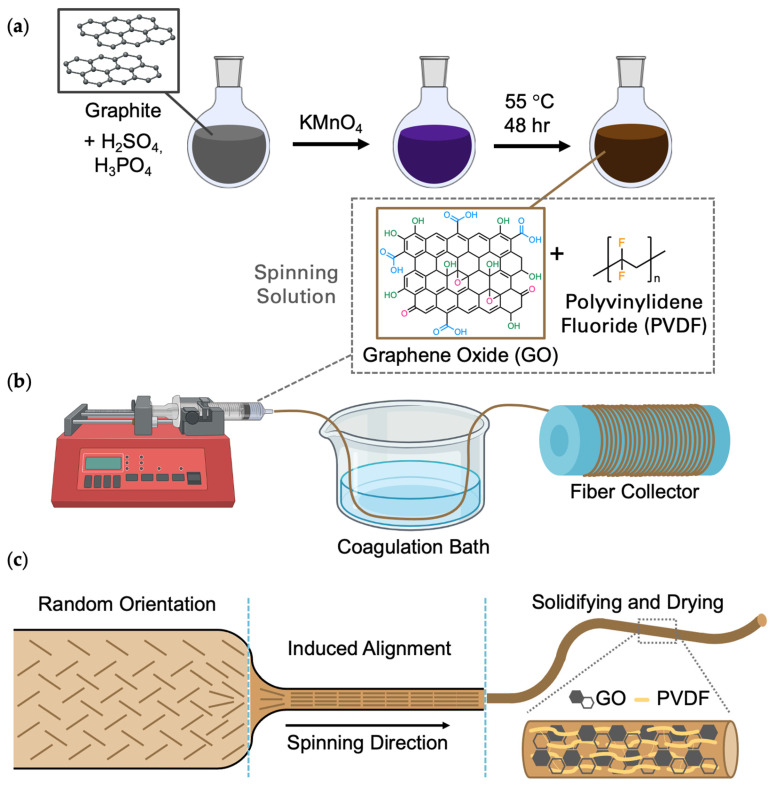
(**a**) Schematic depicting the synthesis of graphene oxide by a modified Hummers’ method; (**b**) Wet-spinning set-up for fabrication of fibers; (**c**) Evolution of fiber formation during the wet-spinning process.

The synthesis of GO via a modified Hummers’ method and the subsequent fabrication of GO-PVDF fibers through wet-spinning techniques are illustrated in Figure 1. The prepared GO nanoflakes are dispersed in a spinning solution containing PVDF (Figure 1a). The wet-spinning process involves extruding the composite solution into a coagulation bath containing the non-solvent and drawing the thread-like material from the bath onto a collector to form meters-long continuous fibers (Figure 1b). The GO flakes are initially randomly distributed in the polymer solution (Figure 1c). Upon injection into the bath, the GO-polymer coagulates due to the solvent/non-solvent exchange. This solvent exchange brings about the phase change that incites the GO-polymer to assemble into the fiber formation. The directional pull from rapid drawing induces the alignment of GO to be parallel with the fiber axis [28]. The stacking of the GO nanosheets within the fiber matrix, pertaining to the dopant concentration, is among the many parameters which affect the fiber morphology [29].

### 3.2. Surface Morphology

#### 3.2.1. Optical Microscopy

**Figure 2:**
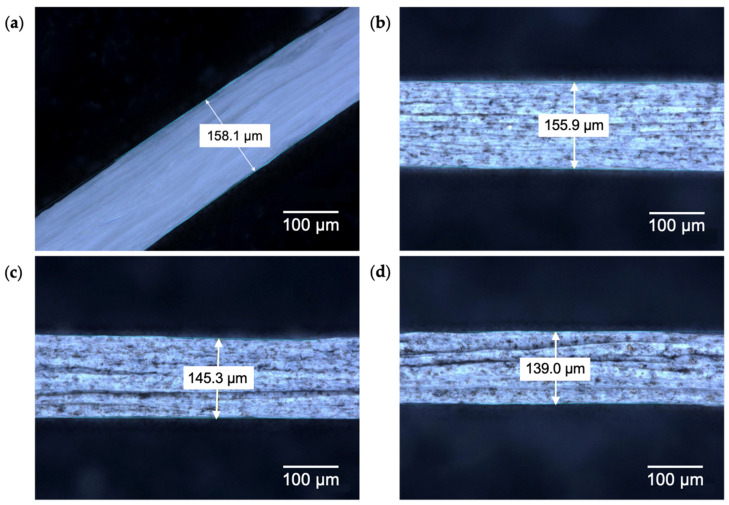
OM images of wet-spun (**a**) undoped PVDF, (**b**) 1 wt.% GO-PVDF, (**c**) 2 wt.% GO-PVDF, and (**d**) 3 wt.% GO-PVDF fibers.

Morphology studies were first carried out through optical microscopy (OM). Images reveal the morphological evolution of the wet-spun GO-PVDF fibers as a function of GO loading, including from 0 wt.%, 1 wt.%, 2 wt.%, and 3 wt.% (Figure 2). The undoped PVDF fibers exhibit a uniform smooth surface, indicative of homogeneity in fiber formation in the absence of dopant (Figure 2a). Upon the incorporation of 1 wt.% GO, an increase in fiber dimensionality is observed, with fibers appearing bulkier and exhibiting surface texture, suggesting the successful integration and dispersion of GO within the polymer matrix (Figure 2b). At 2 wt.% GO loading, the fibers demonstrate pronounced morphological variations, including surface grains and irregularities (Figure 2c). This distinction becomes more apparent at 3 wt.% GO, where the fibers show significant surface defects with the appearance of creased structures along the fiber axis (Figure 2d). These features are a result of interfacial interactions between GO and PVDF, where heterogeneous bonding increases with the addition of dopant [30]. Despite higher GO loadings leading to the emergence of surface irregularities, the fiber diameter remains largely consistent, suggesting that the wet-spinning process is sufficiently controlled and disassociates morphological modifications from macroscopic changes.

The distribution of GO throughout the fiber matrix is demonstrated across all doped fibers. However, GO nanoflakes appear to agglomerate within the fiber, leading to non-uniform dispersity of GO on the surface of the fiber. It has been observed that, owing to their strong adhesion, GO sheets have a propensity to aggregate and thus distort out-of-plane, thereby being susceptible to buckling and rippling [31]. As a result, regions with locally low dopant content are formed along the fiber, creating micro-voids that manifest as the observed surface texture. During the solidification stage of the fiber fabrication process, this behavior may be exacerbated as the GO sheets fold and deform due to solvent evaporation, prompting the formation of a wrinkled morphology characteristic of graphene-based fibers [28].

#### 3.2.2. Scanning Electron Microscopy

**Figure 3:**
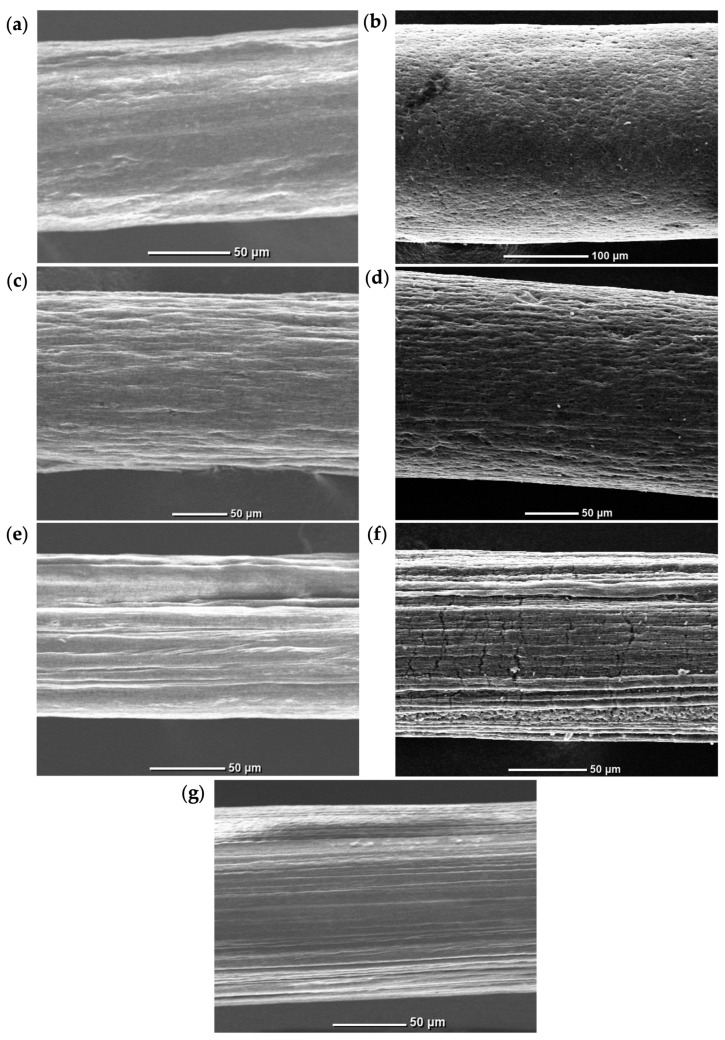
SEM images of wet-spun fibers containing (**a**) 1 wt.% GO, (**b**) 1 wt.% RGO, (**c**) 2 wt.% GO, (**d**) 2 wt.% RGO, (**e**) 3 wt.% GO, (**f**) 3 wt.% RGO, and (**g**) undoped PVDF, respectively.

Scanning electron microscopy (SEM) was employed to further investigate the morphology of wet-spun GO-containing as well as RGO-containing fibers. The SEM images corroborate the observations of OM results by showing the surface features and microstructural modifications induced by GO doping. As the GO loading increases, the fiber texture also increases, and striations in the spinning direction become more prominent on the fiber surface (Figure 3a–c). At the highest loading (Figure 3c), the fibers exhibit the most significantly roughened surface with more pronounced morphological features, specifically larger creases.

Following a chemical reduction, the overall structure of the fibers is retained. At 1 wt.% loading (Figure 3a,d), the same features are observed in GO and RGO fibers, both showing slightly increased surface texture. At 2 wt.% RGO-loaded fibers (Figure 3e), these defects are more prevalent. This is to be expected, as higher GO concentrations lead to greater agglomerations, resulting in an increase in the volume of vacancies throughout the fiber. Prominent changes are exhibited in 3 wt.% loaded fibers after the reduction (Figure 3f). In addition to the presence of wrinkles along the fiber axis, crack-like ridges emerge both parallel and perpendicular on the fiber surface. These deformations can be attributed to shrinkage and stiffening due to the removal of oxygen-containing functional groups from GO during chemical reduction [32]. Within the confinement of the fiber matrix, the reduction of GO causes strain that is released through out-of-plane deformation, which generates fractures at the surface [33].

In comparing the doped fibers to the undoped PVDF fiber, the surface of the undoped fiber appears planar, with the presence of striations parallel to the longitudinal distribution (Figure 3g). These linear ridges are aspects of elongation forces imparted by the spinneret during the wet-spinning process, and are distinctive of wet-spun PVDF fibers [34]. The fiber containing only PVDF had an average diameter of 137.4 μm. The GO-loaded fibers exhibited diameters of 122.3 μm, 157.3 μm, and 123.3 μm for 1, 2, 3 wt.% loadings, respectively, while the RGO-loaded fibers displayed diameters of 125.4 μm, 161.7 μm, 126.0 μm for the same concentrations. The fiber diameters measurements obtained from SEM were relatively consistent across all formulations, with an average of 136.2 ± 15.87 μm, and were in agreement with those determined from OM.

#### 3.2.3. Atomic Force Microscopy

**Figure 4:**
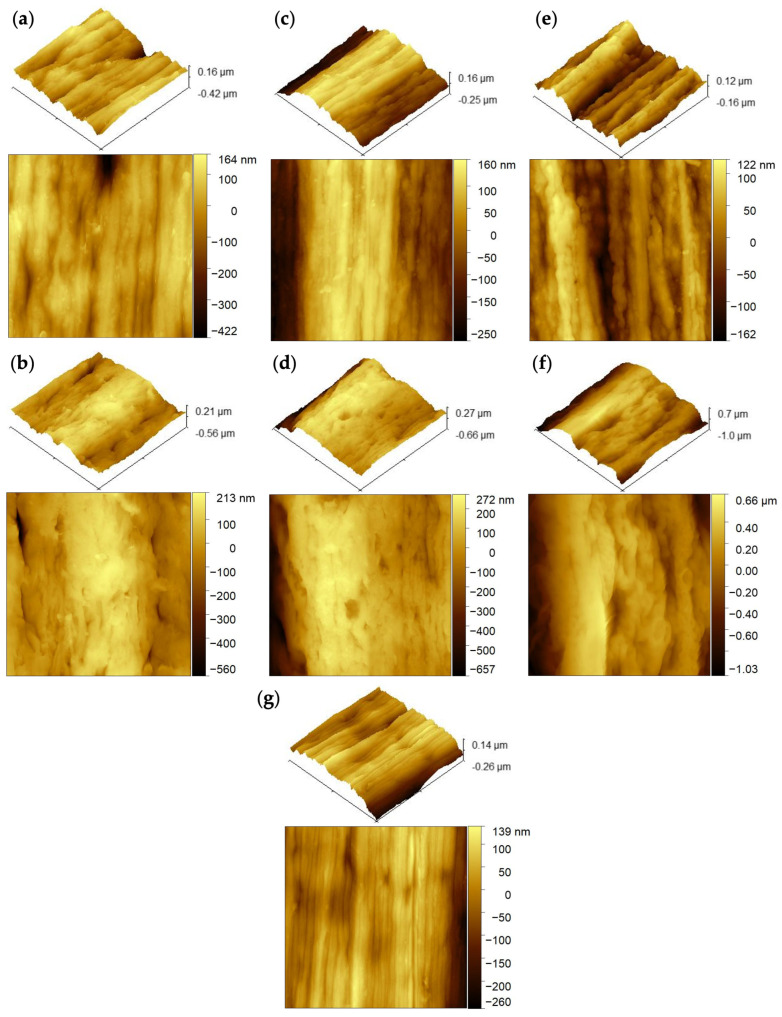
AFM images of wet-spun fibers containing (**a**) 1 wt.% GO, (**b**) 1 wt.% RGO, (**c**) 2 wt.% GO, (**d**) 2 wt.% RGO, (**e**) 3 wt.% GO, (**f**) 3 wt.% RGO, and (**g**) undoped PVDF, respectively.

Atomic force microscopy (AFM) was employed to provide further insight into the surface morphology and nanoscale topography of the GO- and RGO-loaded PVDF fibers. AFM analysis enabled evaluation of surface roughness as a function of graphene loading (1, 2, and 3 wt.%) of GO and RGO. Topographical images revealed increasingly pronounced surface features with higher graphene content, characterized by depth of ridges and more heterogeneous surface textures (Figure 4).

Average surface roughness (R_a_) consistently increased with increasing loading, for both GO- and RGO-containing fibers. At 1 wt.% loading (Figure 4a,b), the R_a_ was measured to be 51.86 ± 4.98 nm for GO fibers and increased to 80.18 ± 3.48 nm following reduction to RGO, indicating that the chemical reduction enhanced surface texturing. A similar trend was observed at 2 wt.% loading (Figure 4c,d), where roughness values increased from 89.20 ± 6.12 nm for GO fibers to 129.2 nm for RGO fibers. The most significant roughening occurred at 3 wt.% loading (Figure 4e,f), where the R_a_ reached 135.6 ± 14.14 nm for GO fibers and further increased to 236.6 ± 17.14 nm upon reduction. At this concentration, the fibers exhibited more distinct and deeper surface ridges, suggesting that higher graphene content amplifies nanoscale surface deformation and structural heterogeneity.

Notably, all graphene-containing fibers exhibited substantially higher roughness compared to the undoped PVDF fiber, which displayed an average R_a_ of only 47.86 ± 3.39 nm (Figure 4g). This contrast highlights the effect of graphene incorporation on fiber surface topology. The improvement in roughness with dopant concentration and chemical treatment suggests that graphene loading and reduction can be strategically exploited to tailor surface morphology. These results suggest that the surface morphology of graphene-enhanced fibers is tunable through controlled variation of GO content and reduction, providing a route to engineer fiber surface properties for targeted composite applications.

### 3.3. Surface Area

#### Brunauer–Emmett–Teller

To further examine the relationship between surface roughness and surface area, Brunauer–Emmett–Teller (BET) analysis was conducted on undoped PVDF, GO-loaded, and RGO-loaded fibers. Given that graphene materials themselves are renowned for high specific surface area, that increased surface roughness is often associated with enhanced surface area [35,36], and that graphene-based materials are inherently characterized by high specific surface area [37], specific surface area measurements were used to assess whether the morphological changes induced by graphene incorporation denote differences in surface area.

**Figure 5:**
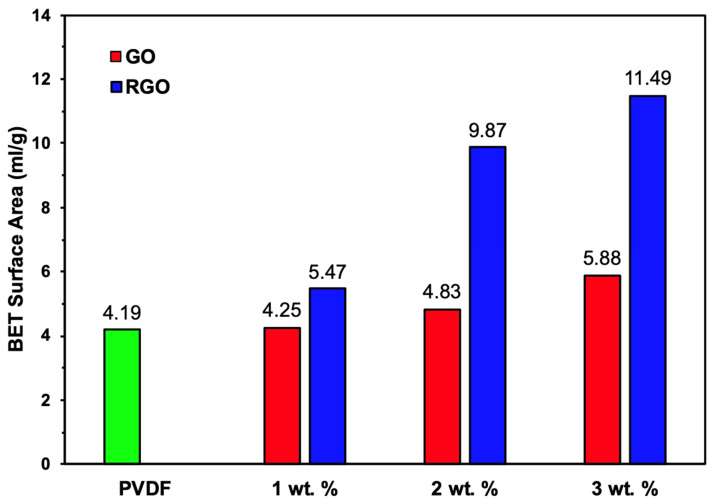
BET specific surface area analysis for undoped PVDF, 1 wt.% GO- and RGO-loaded fibers, 2 wt.% GO- and RGO-loaded fibers, and 3 wt.% GO- and RGO-loaded fibers.

The BET results revealed a clear enhancement in surface area for both GO- and RGO-containing fibers (Figure 5), consistent with the trends observed in AFM roughness measurements. Undoped PVDF fibers exhibited the lowest surface area, reflecting their relatively smooth and homogeneous surface morphology. Upon incorporation of GO, the surface area increased as graphene loading rose from 1 wt.% to 2 wt.% to 3 wt.%, indicating that the introduction of graphene nanosheets effectively modified the fiber surface.

A further enhancement in surface area was observed following the reduction of GO to RGO at each loading. This increase can be attributed to the structural rearrangement and buckling of graphene sheets during the reduction process, which likely creates additional nanoscale defects and wrinkles that contribute to greater surface accessibility. The most significant surface area enhancement was recorded with the 3 wt.% RGO-loaded fiber, corresponding to the highest roughness values measured by AFM. At this concentration, the surface area of the GO-loaded fiber was determined to be 5.88 mL/g and nearly doubled to 11.49 mL/g following the reduction to RGO. These results support the notion that surface morphology and surface area of graphene-enhanced fibers can be effectively tuned through controlled dopant loading, as well as chemical state (oxidized and reduced). Such tunability is advantageous for applications where high surface area and tailored structural properties are critical, including functional composite and sensing systems [38,39].

The surface morphology and BET results collectively reveal a relationship between graphene loading and reduction, as well as surface roughness and surface area. OM and SEM images show that the incorporation of GO and RGO introduced surface defects and dimensionality, leading to the emergence of wrinkle and ridge features. These observations are consistent with the AFM analysis, exhibiting an increase in the R_a_ by 183% for 3 wt.% GO and further increased by 394% for 3 wt.% RGO, relative to the undoped fibers. These results corroborate the BET analysis, demonstrating that the specific surface area increased by 40% with 3 wt. GO % and was further increased by 174% after reduction to RGO, relative to the PVDF fibers. These findings broadly indicate that graphene incorporation and subsequent reduction enhance the fiber morphology, promoting structural complexity and roughened surface that generate greater surface area.

### 3.4. Chemical Properties

#### 3.4.1. Fourier Transform Infrared Spectroscopy

**Figure 6:**
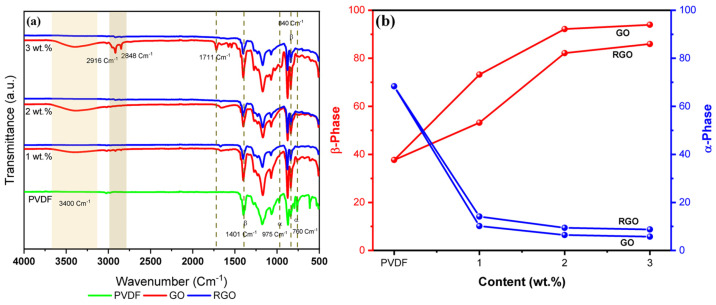
(**a**) ATR-FTIR spectra and (**b**) ratio of β-phase and α-phase.

Fourier transform infrared (FTIR) spectroscopy was employed to examine the chemical structure of undoped PVDF, GO-loaded PVDF fibers, and RGO-loaded PVDF fibers, with particular emphasis on functional group evolution following graphene incorporation and reduction. In Figure 6a, the FTIR spectrum of PVDF, the peaks located at 765, 798, and 975 cm^−1^ are characteristic of the α phase, while those observed at 840, 1071, 1234, 1278, and 1401 cm^−1^ correspond to the β phase [40]. Compared to pure PVDF, the intensities of the absorption bands corresponding to the α phase, located at 765, 798, and 975 cm^−1^, are greatly attenuated, or even completely suppressed, in PVDF fibers loaded with GO and RGO. Conversely, a new peak appears at 840 cm^−1^, whose intensity increases further with the added GO and RGO. This phenomenon is probably attributed to the fact that GO and RGO promote an increase in the proportion of the piezoelectric β phase of PVDF. Indeed, the π electron cloud of the sp^2^ carbon atoms present in GO and RGO can interact with the –CH_2_ dipoles of the PVDF chains, inducing a local organization of all-trans conformations. This interaction favors the stabilization and formation of the polar β phase of PVDF [41]. In addition, the spectrum of PVDF alone exhibited characteristic C–F stretching vibrations at approximately 1270 cm^−1^, which were consistently present in all GO- and RGO-containing fibers. The persistence of this peak across all samples confirms that the PVDF polymer matrix remained intact throughout fiber fabrication, graphene incorporation, and chemical reduction, and that graphene doping did not chemically degrade or alter the structure of PVDF. The small absorption bands at 2848 cm^−1^ and 2916 cm^−1^ correspond to the symmetric CH_2_ and CH groups and asymmetric stretching vibrations, respectively.

In contrast, distinct spectral differences were observed between GO- and RGO-loaded fibers, providing evidence of successful graphene incorporation and subsequent reduction. GO-containing fibers displayed a broad absorption band around ~3400 cm^−1^, corresponding to O–H stretching vibrations associated with hydroxyl groups in GO. This band was absent in RGO-loaded fibers, indicating that these oxygen-containing groups were effectively significantly diminished or removed during the reduction process. Additionally, GO-loaded fibers exhibited a characteristic adsorption near ~1711 cm^−1^, attributed to C=O stretching from carbonyl or carboxyl groups in GO. This peak was not observed in RGO-containing fibers, further confirming the successful chemical reduction of GO to RGO and the removal of oxygenated functionalities.

To better understand, both intuitively and quantitatively, the effect of GO and RGO loading on the proportions of the α (nonpolar) and β (electroactive) phases of PVDF, the relative contents of these phases were evaluated. This analysis was performed assuming that spectral absorption obeys Lambert–Beer’s law, thus allowing the fractions of phases α and β to be determined from Equation (1). The results in Figure 6b indicate that the α and β (F(β)) phase contents of pure PVDF were 66.39% and 35.68%, respectively. It can be observed that fibers containing GO and RGO show a reduction in the α-phase content, accompanied by a significant rise in the β-phase content. For example, the addition of 3 wt.% GO or RGO led to a decrease in the α-phase content (5.77% and 8.37%, respectively), while a marked increase in the β-phase content was observed, reaching 93.95% and 85.83%, respectively. These results highlight that incorporating GO or RGO increases not only the β-phase content of PVDF but can also serve as a key factor in the polarization of the material, thereby enhancing its piezoelectric properties and paving the way for a wide range of applications. Moreover, fibers containing GO have a higher β-phase content than those loaded with RGO, which is attributed to the presence of functional oxygen-containing groups that promote interactions and dipole alignment within the PVDF.

#### 3.4.2. Raman Spectroscopy

Raman spectroscopy was performed to complement the FTIR findings by confirming the structural characteristics and defect evolution of graphene within the GO- and RGO-loaded PVDF fibers. In Raman spectroscopy, the D band (~1350 cm^−1^) is associated with defects and sp^3^-hybridized carbon, while the G band (~1580–1600 cm^−1^) corresponds to the in-plane vibration of sp^2^-bonded carbon atoms in graphitic domains [42,43]. The intensity ratio of the D band to the G band (I_D_/I_G_) is commonly used as an indicator of defect density and the degree of structural disorder in graphene and its derivatives [44].

**Figure 7:**
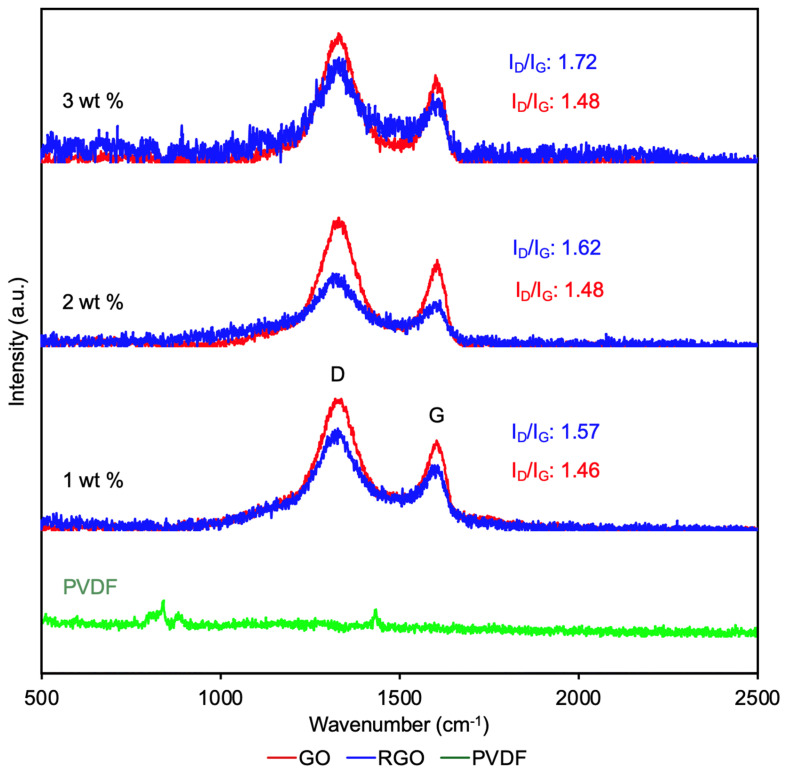
Raman spectra of 1 wt.% GO- and RGO-loaded fiber, 2 wt.% GO- and RGO-loaded fiber, 3 wt.% GO- and RGO-loaded fiber, and undoped PVDF fiber.

For fibers containing 1 wt.% graphene, the I_D_/I_G_ ratio increased from 1.46 in GO fibers to 1.57 following reduction to RGO (Figure 7). A similar behavior was observed at 2 wt.% loading, where the I_D_/I_G_ ratio increased from 1.48 for GO fibers to 1.62 for RGO fibers. At the highest loading of 3 wt.%, the I_D_/I_G_ ratio further increased from 1.48 in GO fibers to 1.72 in RGO fibers. Across all concentrations, the systematic increase in I_D_/I_G_ upon reduction indicates an increase in defect density within the graphene structure.

This increase in I_D_/I_G_ following reduction is consistent with the removal of oxygen-containing functional groups from GO, which disrupts the sp^2^ carbon lattice and generates additional structural defects and fragmentation in graphitic domains [45,46]. Rather than indicating poorer material quality, this trend is typical for the GO-to-RGO conversion and reflects the emergence of new defect sites and edge structures during chemical reduction. The gradual rise in I_D_/I_G_ with increasing graphene loading, particularly in RGO fibers, suggests that higher concentrations promote greater structural disorder and disruption of graphitic domains within the composite matrix.

### 3.5. Mechanical Properties

**Figure 8:**
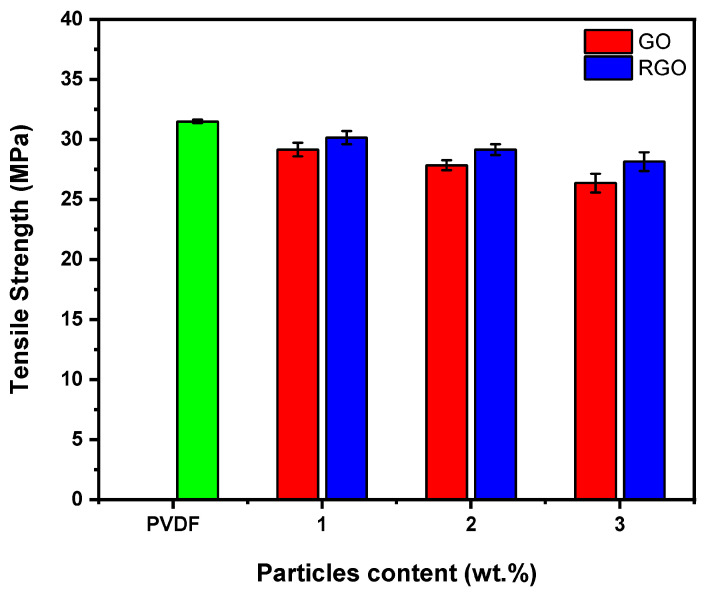
Tensile strength of 1 wt.% GO- and RGO-loaded fiber, 2 wt.% GO- and RGO-loaded fiber, 3 wt.% GO- and RGO-loaded fiber, and undoped PVDF fiber.

Mechanical properties of fibers containing 1, 2, and 3 wt.% GO-PVDF and RGO-PVDF were evaluated by tensile testing. Figure 8 illustrates the tensile strength of GO-PVDF and RGO-PVDF fiber blends containing various loading contents. The GO- and RGO-loaded fibers show a slight depletion in tensile strength, reaching about 16% and 12%, respectively, compared to undoped PVDF fibers. For instance, the tensile strength of PVDF, which was initially 31.49 ± 0.15 MPa, decreases to 26.36 ± 0.74 MPa and 28.15 ± 0.79 MPa after adding 3 wt.% of RGO and GO, respectively. This decrease is probably attributed to microstructural changes induced by the increase in filler content. With increasing filler content, inhomogeneities or regions of discontinuity may be present in the matrix, as observed by SEM [47,48,49]. The delamination observed may be linked to certain discontinuities seen in the SEM images. These structural irregularities can compromise the overall cohesion of the material and limit the efficiency of mechanical stress transfer within the composite structure. Under stress, these zones can cause cracks to form and propagate within the fibers, thereby contributing to a reduced tensile strength. Moreover, PVDF-based fibers reinforced with RGO exhibit moderately higher tensile strengths than those containing GO, which can be explained by better dispersion of RGO within the polymer matrix, combined with more moderate agglomeration, leading to more effective interfacial adhesion compared to GO.

**Figure 9:**
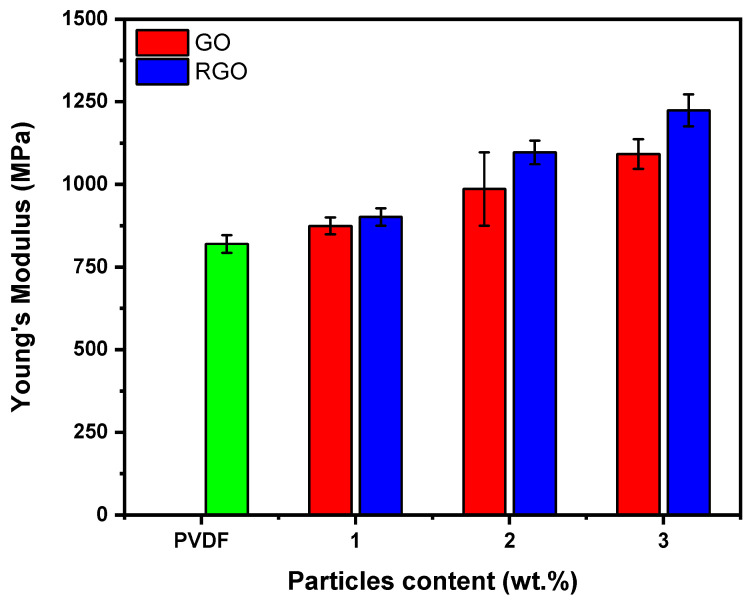
Young’s modulus of 1 wt.% GO- and RGO-loaded fiber, 2 wt.% GO- and RGO-loaded fiber, 3 wt.% GO- and RGO-loaded fiber, and undoped PVDF fiber.

Figure 9 shows the Young’s modulus of PVDF-based fibers loaded with different contents of GO and RGO. As expected, the doped fibers have higher Young’s modulus values compared to the undoped PVDF fibers. For instance, the Young’s modulus of PVDF fibers, which was initially 820 ± 26.74 MPa, increases significantly to 1091.74 ± 44.73 MPa and 1224 ± 48.49 MPa after adding 3 wt.% of GO and RGO, corresponding to gains of 33% and 49%, respectively. This behavior is frequently observed when rigid particulate loads are added to more flexible thermoplastic matrices, as the reinforcing loads tend to reduce the motion of the polymer matrix chains under tensile stress [50,51,52]. In addition, RGO-loaded PVDF fibers proved to increase the Young’s modulus more than GO-loaded PVDF fibers due to the higher intrinsic stiffness and specific surface area of RGO. The latter exhibits better dispersion within the PVDF matrix, thus promoting effective interfacial adhesion and optimal stress transfer, which leads to increased material stiffness. A larger surface area provides more interfacial interaction between RGO and PVDF matrix, which can facilitate more efficient stress transfer throughout the composite fiber. Thus, fibers containing graphene exhibit increased stiffness, as reflected in the higher Young’s modulus measured for GO- and RGO-doped fibers compared to undoped PVDF.

**Figure 10:**
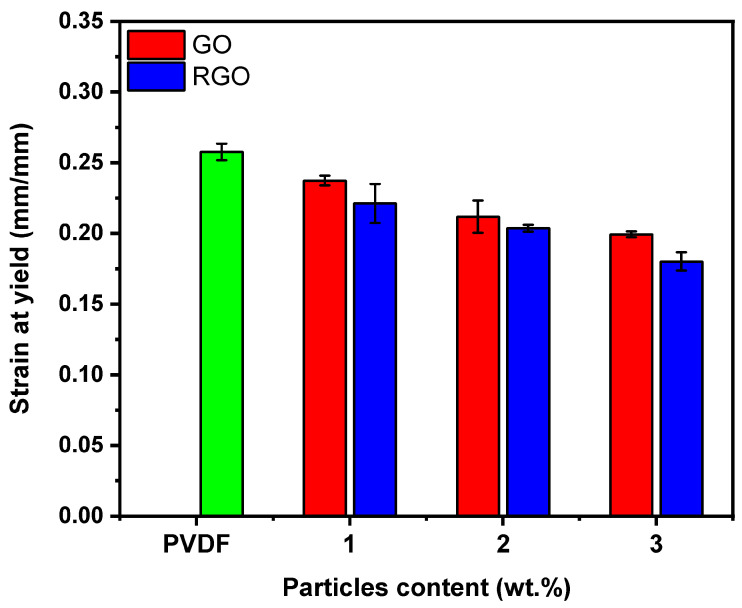
Strain at yield of 1 wt.% GO- and RGO-loaded fiber, 2 wt.% GO- and RGO-loaded fiber, 3 wt.% GO- and RGO-loaded fiber, and undoped PVDF fiber.

In general, studies of the tensile properties of loaded composites show that high Young’s modulus values are commonly associated with low strain at yield. Figure 10 displays the evolution of strain at yield for PVDF-based fibers as a function of GO and RGO concentration. As expected, the strain at yield of pure PVDF, which was initially 0.257 ± 0.006 mm/mm, has decreased after loading the fillers GO and RGO. Strain at yield decreases to values of 0.0199 ± 0.02 and 0.18 ± 0.0065 mm/mm after adding 3 wt.% of GO and RGO, respectively, which can be attributed to the restricted deformability of the polymer matrix, linked to its reduced ability to absorb plastic energy [53]. Furthermore, adding rigid fillers to flexible thermoplastic polymers tends to hinder the motion of the matrix chains and limit their relative slippage, resulting in low deformation at the elastic limit. A further reason for the lower ductility of reinforced composites could be caused by the presence of discontinuities in the matrix [54]. It is well established that the formation of voids or defects during tensile deformation is closely linked to the strength of the interface between the polymer matrix and the reinforcing particles. Thus, insufficient affinity promotes the appearance of voids or tearing phenomena, leading to local deformation of the polymer and facilitating the nucleation and propagation of microcracks within the matrix [55]. Even though the incorporation of GO and RGO led to an increase in Young’s modulus and a corresponding decrease in strain at yield, the tensile strength exhibited only a reduction of approximately 12% at the highest 3 wt.% RGO loading, suggesting that the fibers retain substantial load-bearing capability despite augmented stiffness.

In order to assess the effect of load types and their content on mechanical properties, a two-way analysis of variance (ANOVA) was conducted using a significance level of 5%. The *p*-value, generated by ANOVA and obtained from the F-statistic (ratio of group mean squares to within-group mean squares), was used to decide whether to refute the null hypothesis or not [56]. Thus, at a 5% significance level, a *p* < 0.05 indicates a statistically significant effect of the factor considered, while a *p* > 0.05 suggests no significant effect. Table 2 presents the results from a two-way ANOVA analysis, illustrating the influence of load type (GO and RGO), load content (1–3 wt.%), and their interaction on tensile parameters, namely tensile strength, Young’s modulus, and strain at yield, at a significance level of α = 0.05. For tensile strength, both the type of load (F = 45.52; *p* = 2.05 × 10^−5^) and the load content (F = 46.85; *p* = 2.14 × 10^−6^) showed statistically significant effects, highlighting that these results suggest that not only the nature of the filler but also its concentration has a marked impact on the overall behavior of the material. Nevertheless, interaction between load type and content was not significant (*p* = 0.308), implying that effects of increased loading content follow a similar trend for GO- and RGO-loaded systems. A similar trend was observed for Young’s modulus, showing that load type had a significant effect on system stiffness (F = 38.25, *p* = 4.69 × 10^−5^), while load content was even more pronounced (F = 53.96, *p* = 1.00 × 10^−6^). Absence of statistically significant interaction (*p* = 0.839) would indicate that an effect to reinforce increased load content is consistent whether GO or RGO is used, confirming that stiffness improvement is governed primarily by load content rather than combined effects of type and content load. With regard to strain at yield, both load types (F = 29.66; *p* = 1.49 × 10^−4^) and load content (F = 73.33; *p* = 1.87 × 10^−7^) were found to have a statistically significant effect on system ductility. However, no significant interaction effect was observed (*p* = 0.259), suggesting that the drop in ductility associated with increased load content occurs similarly in GO- and RGO-based systems.

To contextualize the mechanical performance of the graphene-enhanced PVDF fibers, the tensile properties obtained in this work were compared with previously reported PVDF-based fibrous composites employing a range of dopants and fabrication techniques in Table 3. The GO- and RGO-loaded fibers fabricated via wet-spinning exhibit tensile strengths of 26.36 MPa and 28.15 MPa, with corresponding Young’s moduli of 1092 MPa and 1224 MPa, respectively. While these values have not reached those reported for certain electrospun or dry-jet wet-spun composites, they fall well within the range of mechanical compliancy for fibrous materials, specifically for those designed for flexible and wearable electronic applications [11,20].

Many reported systems achieving higher tensile strength rely on more complex fabrication routes, whereas the wet-spinning approach in this work represents a facile and scalable fabrication method that uses minimal equipment. These GO- and RGO- enhanced wet-spun PVDF fibers offer a balanced combination of mechanical robustness and structural complexity. The integration of tunable morphology, enhanced surface area, and potential higher piezoelectricity positions these fibers as promising materials for wearable sensors and flexible electronic systems.

## 4. Conclusions

This study presents a comprehensive morphological investigation of GO- and RGO-doped wet-spun PVDF fibers, establishing the relationships between fiber morphology and mechanical properties. By elucidating these structure–property correlations, this work provides a foundation for morphology-oriented design principles to enable targeted performance in graphene-enhanced fibrous composites. In this work, GO- and RGO-enhanced PVDF fibers were successfully fabricated using a wet-spinning process, and their structural, chemical, surface, and mechanical properties were studied. Extensive structural characterization and mechanical testing demonstrated that graphene incorporation and subsequent reduction enable effective tuning of fiber morphology while maintaining overall mechanical integrity.

SEM and AFM results revealed that increasing GO and RGO loading led to pronounced increases in surface dimensionality and roughness, with the average roughness increasing by nearly fivefold upon incorporation of 3 wt.% RGO compared to undoped fibers. Consistent with these observations, BET analysis showed a moderate increase in specific surface area with 3 wt.% GO addition, while chemical reduction to RGO further amplified this effect, resulting in almost double the surface area. ATR-FTIR confirmed the successful incorporation of graphene derivatives and effective reduction of GO while preserving the PVDF matrix. The results indicate that graphene incorporation not only enables control over fiber structural and surface features but also influences the crystalline phase of PVDF. The ATR-FTIR data also provided evidence that both GO- and RGO-doped fibers exhibited induction of the piezoelectric β-phase, with the highest β-phase content observed for fibers containing 3 wt.% GO. Tensile testing unveiled that graphene incorporation increased stiffness and reduced ductility. However, both GO- and RGO-loaded fibers retained sufficient flexibility and mechanical robustness, positioning their performance within the range of PVDF-based fibrous composites reported for wearable and flexible electronic applications.

Overall, this study demonstrates that graphene doping offers a versatile and tunable strategy for engineering PVDF fiber morphology with only marginal compromise in mechanical compliance. Further exploration will focus on evaluating the piezoelectric behavior of these fibers and their functional performance in device architectures, particularly under dynamic mechanical loading conditions. The combination of surface morphology modulation, enhanced surface area, and the potential for piezoelectric behavior highlights the potential of these graphene-enhanced fibers for advanced wearable sensing and flexible electronic applications.

## Figures and Tables

**Table 1:** Composition of wet-spinning solution.

GO-PVDF Spinning Solution	GO	PVDF	DMF
0 wt.%	0.00 g	1.50 g	3.50 g
1 wt.%	0.05 g	1.50 g	3.45 g
2 wt.%	0.10 g	1.50 g	3.40 g
3 wt.%	0.15 g	1.50 g	3.35 g

**Table 2:** Two-way ANOVA results the effects of load type (GO and RGO), load content (1–3 wt.%), and their interaction on tensile parameters (α = 0.05).

Property	Factor	F-Value	*p*-Value	Significance (α = 0.05)
Tensile strength	Load type (GO and RGO)	45.51925	$2.05333\times 10^{-5}$	Significant
	Load content (wt.%)	46.84988	$2.14113\times 10^{-6}$	Significant
	Interaction	1.29825	0.30874	Not Significant
Young’s modulus	Load type (GO and RGO)	38.24888	$4.69452\times 10^{-5}$	Significant
	Load content (wt.%)	53.96069	$1.00394\times 10^{-6}$	Significant
	Interaction	0.17794	0.83916	Not Significant
Strain at yield	Load type (GO and RGO)	29.65641	$1.48674\times 10^{-4}$	Significant
	Load content (wt.%)	73.33134	$1.87171\times 10^{-7}$	Significant
	Interaction	1.51632	0.25875	Not Significant

**Table 3:** Comparison of different dopants in PVDF-based fibrous composites for wearable electronic applications.

Dopant	Fabrication Method	Tensile Strength	Young’s Modulus	Targeted Application	Ref.
3 wt.% GO	Wet-spinning	26.36 MPa	1092 MPa	This work	
3 wt.% RGO	Wet-spinning	28.15 MPa	1224 MPa		
2 wt.% GO	Dry-jet wet-spinning	394 MPa	4600 MPa	—	[20]
0.7 wt.% GO	Electrospinning	9 MPa	—	Pressure sensor, transducer	[11]
0.03 wt.% MWCNT	Electrospinning	48.17 MPa	1390 MPa	Wearable piezoelectric sensor	[57]
0.002 wt.% MWCNT	Electrospinning	906 MPa *	—	Wearable piezoelectric device	[58]
2.5 wt.% BNNT	Electrospinning	18.10 MPa	—	Wearable piezoelectric sensor	[59]
4 wt.% WO_3_	Electrospinning	4 MPa	—	Piezoelectric nanogenerators	[60]

* Value converted from 0.58 g/den.

## Data Availability

The original contributions presented in this study are included in the article. Further inquiries can be directed to the corresponding author.
